# International validation of quality indicators for evaluating priority setting in low income countries: process and key lessons

**DOI:** 10.1186/s12913-017-2360-7

**Published:** 2017-06-19

**Authors:** Lydia Kapiriri

**Affiliations:** Department of Health, Aging and Society, McMaster University, 1280 Main Street West, Hamilton, ON Canada

**Keywords:** Priority setting, Evaluation, Framework, Validation, Low income countries

## Abstract

**Background:**

While there have been efforts to develop frameworks to guide healthcare priority setting; there has been limited focus on evaluation frameworks. Moreover, while the few frameworks identify quality indicators for successful priority setting, they do not provide the users with strategies to verify these indicators. Kapiriri and Martin (Health Care Anal 18:129-147, 2010) developed a framework for evaluating priority setting in low and middle income countries. This framework provides BOTH parameters for successful priority setting and proposes means of their verification. Before its use in real life contexts, this paper presents results from a validation process of the framework.

**Methods:**

The framework validation involved 53 policy makers and priority setting researchers at the global, national and sub-national levels (in Uganda). They were requested to indicate the relative importance of the proposed parameters as well as the feasibility of obtaining the related information. We also pilot tested the proposed means of verification.

**Results:**

Almost all the respondents evaluated all the parameters, including the contextual factors, as ‘very important’. However, some respondents at the global level thought ‘presence of incentives to comply’, ‘reduced disagreements’, ‘increased public understanding,’ ‘improved institutional accountability’ and ‘meeting the ministry of health objectives’, which could be a reflection of their levels of decision making. All the proposed means of verification were assessed as feasible with the exception of meeting observations which would require an insider. These findings results were consistent with those obtained from the pilot testing.

**Conclusions:**

These findings are relevant to policy makers and researchers involved in priority setting in low and middle income countries. To the best of our knowledge, this is one of the few initiatives that has involved potential users of a framework (at the global and in a Low Income Country) in its validation. The favorable validation of all the parameters at the national and sub-national levels implies that the framework has potential usefulness at those levels, as is. The parameters that were disputed at the global level necessitate further discussion when using the framework at that level. The next step is to use the validated framework in evaluating actual priority setting at the different levels.

**Electronic supplementary material:**

The online version of this article (doi:10.1186/s12913-017-2360-7) contains supplementary material, which is available to authorized users.

## Background

Priority setting still remains a big challenge for policy makers in low income countries [1]. The priority setting contexts which are marked with extreme resource constraints, political instability, and limited institutional capacity play a critical role with regards to how priorities are set and if they are ever implemented. [2–7] Priority setting has been found to provide guidance in situations where there is a gap between what should be done (for example according to the health policy) and the available resources. Priority setting is important in policy formulation, as well as implementation. While there has been progress in developing frameworks to *guide* priority setting for health interventions in Low income countries (LIC), limited efforts have been devoted to developing corresponding evaluation frameworks and/or quality indicators to enable policy makers to evaluate priority setting. Moreover, while factors such as stakeholder engagement (and the differences in their decision making powers, and their legitimacy), accountability, the use of evidence, and the role of contextual factors are well described in the literature, the degree to which relevant stakeholders perceive them as relevant when evaluating priority setting processes has not been established. [8–16] This paper fills this gap in the literature by presenting findings from a study which validated a framework developed to evaluate priority setting in low income countries.

The literature presents previous efforts to develop frameworks for evaluating successful priority setting. Based on both empirical and theoretical information, Gibson and colleagues [13], [14] identified quality indicators for health intervention priority setting in health management organizations and hospitals in Canada. Sibbald and colleagues [18, 19] also identified and pilot-tested similar parameters for successful priority setting, mainly within the context of high income countries. More recently, Barasa and colleagues [19], based on a synthesis of current literature, proposed a conceptual framework for evaluating macro and meso-evel priority setting, whose parameters were similar to those identified by Gibson et al. [13] and Sibbald et al. [18]. While Barasa’s paper reviewed all current literature, Gibson and Sibbald’s studies focused mainly on priority setting in high income countries. In response to the uniqueness of priority setting in low income countries, Kapiriri and Martin [20] developed a framework for evaluating priority setting in low income countries. Theoretically, the framework was informed by the evaluation literature, as well as Daniel’s ‘Accountability for Reasonableness’ framework (which focuses on procedural justice) [21], the work of Gibson and colleagues [18] and Sibbald and colleagues [17, 18] coupled with interviews with experts at the global level, and their experience with priority setting in low income countries. The framework recognizes that priority setting is both a technical and a political process and hence catered for both by considering parameters such as the use of evidence and explicit tools (technical), as well as the role of stakeholders and the politics of engagement (political). In relationship to these parameters, the framework also recognizes the unique priority setting context of low income countries and deliberately considers the contextual factors that may affect successful priority setting in low income countries, in addition to responding to some of critical issues that Sibbald and colleagues [18] identified when they pilot tested their framework. Kapiriri & Martin’s framework [20] identifies parameters that are directly related to the priority setting institution, (internal) and those that are beyond the institution (external) parameters. Furthermore, the framework identified immediate and delayed parameters (relating to the time when they can be evaluated) for evaluating priority setting. Recognizing the difficulties that policy makers are faced with when trying to identify the relevant sources of information, the framework also identifies objectively verifiable indicators for each of the parameters and proposes means for verifying the quality indicators. The means of verification proposed in the framework include: observation at decision-making meetings with exit interviews, review of documents (including policy, publications, newspapers, special reports and health facility documents) and conducting public opinion and satisfaction surveys. The framework also recognizes the importance of the broader political, economic and cultural context for the success of any priority setting and identifies relevant contextual factors that should be understood in order to achieve a holistic evaluation on which viable, locally-specific, improvement strategies can be based. These parameters, the indicators and means of verification are summarised in Table 1.

**Table 1:** Parameters for evaluating Priority setting with corresponding means of verification and indicators

	Means of Verification (MOV)	Objectively Verifiable Indicators (OVI)
Immediate Parameters of Successful Priority Setting		
Efficiency of the priority-setting process	Proportion of meeting time spent on PS, number of decisions made on time	Observations/min at meetings, annual budget documents, health system reports
Allocation of resources according to priorities	Degree of alignment of resource allocation and agreed upon priorities, times budget is re-allocated from less prioritized to high prioritized areas, stakeholder satisfaction with the decisions	Annual budget reports, evaluation documents
Stakeholder participation	Number SH participating, number of opportunities each SH gets to express opinion	Observations/min at meetings, media reports, special reports
Use of clear priority setting process/tool/methods	Documented PS process and/or use of Ps framework	Observation/min at meetings, media reports, special reports
Use of evidence	Number of times available data is resourced/number of studies commissioned/existing strategies to collect relevant data	Observations/min at meetings, media reports, special reports
Use of explicit relevant priority setting criteria	Documented/articulated criteria	Observations/min at meetings, media reports, special reports
Publicity of priorities and criteria	Number of times decisions and rationales appear in public documents	Media reports
Functional mechanisms for appealing the decision	Number of decisions appealed, number of decisions revised	Observations/min at meetings, media reports, special reports
Functional mechanisms for enforcement	Number of cases of failure to adhere to priority-setting process reported	Observations/min at meetings, media reports, special reports
Reflection of public values	Number and type of members from the general public represented, how they are selected, number of times they get to express their opinion, proportion of decisions reflecting public values, documented strategy to enlist public values, number of studies commissioned to elicit public values	Observations/min at meetings, study reports, meeting minutes and strategic plans
Increased public awareness of PS	% of public aware of existing PS process	Public awareness study
Increased public confidence and acceptance of decisions	Number of complaints from the public	Reports, minutes from meetings, media reports
Delayed Parameters of Successful Priority Setting		
Increased stakeholder understanding, satisfaction and compliance with the PS process	Number of SH attending meetings, number of complaints from SH, % SH that can articulate the concepts used in PS and appreciate the need for PS	Observations/min at meetings, special reports, SH satisfaction survey, media reports, stakeholder interviews, evaluation reports
Decreased dissentions	Number of complaints from SH	Meeting minutes, media reports
Decreased resource wastage/misallocation	Proportion of budget unused	Budget documents, evaluation reports
Improved internal accountability/reduced corruption	Number of publicized resource allocation decisions	Evaluation reports, stakeholder interviews, media reports
Strengthening of the PS institution	Indicators relating to increased efficiency, use of data, quality of decisions and appropriate resource allocation, % stakeholders with the capacity to set priorities	Training reports, evaluation reports, budget documents
Impact on institutional goals and objectives	% of institutional objectives met that are attributed to the priority setting process	Evaluation reports, special studies
Impact on health policy and practice	Changes in health policy to reflect identified priorities	Policy documents
Achievement of HS goals	% reduction in DALYs, % reduction of the gap between the lower and upper quintiles, % of poor populations spending more than 50% of their income on health care, % users who report satisfaction with the healthcare system	National budget allocation documents, human resources survey reports, Interviews with stakeholders
Improved financial and political accountability	Number of publicized financial resource allocation decisions, number of corruption instances reported, % of the public reporting satisfaction with the process	Reports, media reports, interviews with stakeholders
Increased investment in the health sector and strengthening of the health care system	Proportion increase in the health budget, proportion increase in the retention of health workers, % of the public reporting satisfaction with the health care system	National budget allocation documents, human resources survey reports, interviews with stakeholders, media reportNational budget allocation documents, human resources survey reports, interviews with stakeholders, media report
Contextual Factors Political, Economic, Social and cultural	Relevant contextual factors that may impact priority setting	Follow up intermittent interviews with local stakeholders, systematic longitudinal observations, relevant reports, media

Source: (Kapiriri & Martin, [20])

While Kapiriri and Martin’s [20] framework received input from a wide range of stakeholders during its development, it received limited systematic review by experts in priority setting and practitioners involved in health planning in low income countries. Conceptually robust frameworks, if not validated by the stakeholders who are likely to use them, may be in danger of having limited buy in. This paper responds to this challenge and reports findings from a validation process of Kapiriri and Martin’s [20] framework. The overall goal of this study was to validate the parameters of successful priority setting and their means of verification, at the global level, the national and sub-national (district) levels in Uganda. The specific objectives were to: 1) Determine the degree to which priority setting experts and practitioners thought the quality indicators for successful priority setting identified in the framework were important. 2) Assess which means of verification were considered most feasible. 3) Determine if there are any differences in assessments of the importance of the various parameters between respondents at the different levels

## Methods

The validation process took place between March 2013 and October 2015, and involved structured interviews, a survey with both experts and practitioners at three levels and pilot testing the proposed means of verification. Study population and sampling. We recruited 53 respondents (Table 2). At the global level two thirds of the respondents were researchers, the rest were development assistance partners. Respondents at the national level included policy makers from the Ministry of Health and a couple of researchers. District respondents included members of the District Health Team; which is responsible for planning and allocating resources at the district level.

**Table 2:** Overview of the respondents

Level of decision-making	Number of respondents
Global	17
National	23
District	13
Total	53

*Global Level*: Both researchers and practitioners involved in priority setting were purposefully identified and contacted, via e-mail, to participate in the study. We contacted researchers who have published on priority setting generally and specifically, in low income countries over the past10 years. These were identified through PubMed, using the search words: “priority setting”, and “low income countries”, “developing countries”.To identify respondents from international organizations involved in priority setting in low income countries, we used the internet. Contact information of the officers involved priority setting was obtained from the organizations’ web pages. *National and sub-national levels*: At the national level, after obtaining permission from the relevant authorities, we identified the Ministry of Health officers in charge of health planning as our index respondents. Subsequent respondents within the Ministry were identified using snowball sampling. The main criterion for recruitment was direct involvement in priority setting at the national level. A similar process was used to identify participants at the district level. The district was.

### Data collection

We re-framed the parameters in the framework, and reorganized them into five categories: Health system-, priority setting process-, public & stakeholder -, priority setting institute- and context related parameters (Table 2). Participants were asked to indicate the degree of importance they attributed to a given parameter when evaluating priority setting. The options included: Very important; somewhat important (average importance) and not important. For the means of verification, respondents were asked to indicate how feasible it would be to collect the information necessary for each of the quality indicators. The options here included very easily, somewhat easily, and not easily. For both questions about the quality indicators and the means of verification, participants were provided with space to provide a qualitative explanation for their responses (Additional file 1).

The survey was administered through Survey Monkey to the respondents at the global level. A trained research assistant administered the survey face to face to the respondents at the national and district levels.

*Pilot testing*: Between March 2013-October 2015, two trained research assistants piloted the proposed means of verification to practically assess their feasibility within the Ugandan context.

Broadly, the framework proposed the following means of verification: reviewing documents (including policy documents, minutes from decision making meetings, relevant publications, and newspaper articles), attending Ministry of Health planning and priority setting meetings, exit interviews with stakeholders attending the planning meetings and population surveys.

### Data analysis

Quantitative responses were summarized by simple frequency tabulation. The corresponding qualitative responses were manually analyzed. Since respondents had the option to provide explanations for each of their responses, qualitative responses for each parameter and means of verification were compiled using survey monkey. These were presented in a spreadsheet. We carefully read through the narratives contained in each cell corresponding to a given parameter, identifying and synthesizing recurring themes within the narratives. These were summarized and are presented. Furthermore, relevant supporting quotes were identified from the narratives and are presented in the results section.

## Results

### Validation of the parameters

Table 3 summarizes the relative importance of the parameters for successful priority setting according to the respondents’ level of decision-making.

**Table 3:** Perceived degree of importance of the different parameters for successful priority setting

	Very important			Average importance			Not important		
Respondents’ levels of operation (number of respondents) G = Global; N = National; D = District	G (17)	N (23)	D (13)	G (17)	N (23)	D (13)	G (17)	N (23)	D (13)
Parameters related to the priority setting process									
Increased Efficiency of the process	14	21	13	3	1	0	0	1	0
Use of an explicit framework	8	23	13	9	0	0	0	0	0
Increased use of evidence	15	23	9	2	0	4	0	0	0
Fairer PS process^a^ (stakeholder involvement, Publicity, explicit relevant criteria, appeals, enforcement)	12	22	9	4	1	2	1	0	2
Availability of incentives for implementers to comply	7	16	8	6	6	5	4	1	0
Increased public input and reflection of public values	11	20	12	6	2	1	0	1	0
Increased stakeholder satisfaction	9	22	13	7	1	0	1	0	0
Increased stakeholder understanding of the PS process	8	23	8	8	0	5	1	0	0
Increased compliance with the process	11	20	8	5	2	5	1	1	0
Reduced disagreements	2	21	12	9	1	0	6	1	0
Increased public awareness and knowledge of PS	4	22	12	10	1	1	3	0	0
Parameters related to the priority setting institute									
Strong legitimate PS institutions with capacity and resources to set and implement priorities	13	21	13	4	0	0	0	2	0
Achievement of priority setting institutional objectives	8	21	13	8	0	0	1	2	0
Parameters related to the health system									
Allocation of resources according to priorities	13	23	13	3	0	0	1	0	0
Reduced resource wastage	11	22	13	3	1	0	3	0	0
Improved internal accountability/reduced corruption	7	21	11	7	1	0	3	1	2
Achievement of health system goals	11	23	12	2	0	0	1	0	1
Improved political and financial accountability	9	23	11	7	0	1	1	0	1
Increased Public confidence in the MOH and acceptance of decisions	8	22	12	8	1	1	1	0	0
Increased investment in the health sector and strengthening of the health care system	6	20	10	7	3	1	4	0	2
Parameters related to the priority setting context									
Favorable Political context and will	17	23	13	0	0	0	0	0	0
Favorable economic context	17	23	13	0	0	0	0	0	0
Favorable social- cultural context	17	23	13	0	0	0	0	0	0

^a^Average value calculated

### District level responses

District respondents evaluated all but five quality indicators to be very important when assessing success in health intervention priority setting. Their qualitative explanations related to the fact that there were limited resources which should be allocated efficiently and appropriately using a process with explicit criteria. They also thought that more appropriate and accountable systems would attract more resources to the health sector. Respondents also evaluated all the parameters related to the priority setting process and the capacity of the people involved in priority setting; as well as those related to public and stakeholder related parameters as very important. All district respondents evaluated the political, social, economic and cultural context as very important when evaluating priority setting. For example, with regards to the relevance of cultural contexts, respondents explained that when priorities are not culturally appropriate and deemed inappropriate within the local context, they may not be locally acceptable and hence not successfully implemented. The political and economic contexts were also deemed relevant in that political instability and lack of political will impacts the implementation of priorities. However, political will and its influence on implementation is mediated through the economic context. Respondents also identified additional relevant factors, these included: the physical infrastructure (impacting successful implementation), and the role/impact of natural disasters/emergencies (deviating priorities and resources).

A few parameters, including stakeholder understanding and compliance, increased internal accountability, fair process and incentives were evaluated as not important by 1–2 respondents. Most respondents deemed stakeholder understanding an important parameter of successful priority setting because it impacts acceptability and potential for sustainability;

“…*When stakeholders understand the priority setting process, they can own the projects and leads to sustainability*…”(D3)

*If stakeholders don’t understand the process of priority setting then the process is likely to fail*…”(D2)

However, two respondents argued that stakeholder participation was not an important parameter since stakeholder participation and comprehension of the priority setting process is linked to literacy levels, which is limited in the Ugandan context.

Another parameter which received a varied evaluation at the district level in Uganda was the use of incentives. While most participants agreed that incentives are very important to a successful priority setting process, one respondent argued that stakeholder understanding of the process, may be the best incentive for compliance, since it facilitated stakeholder ownership of the process, and interest in seeing it succeed.

The use of evidence also received a lower rating among a couple of participants. The majority of participants rated it highly because they thought it was important for advocacy, added credibility to the decisions.

“…*In the modern decision-making, it is important to use evidence (empirical) to convincedecision makers*…”(D8)

*“… It even becomes impossible for anyone to challenge you on decisions made*…” (D6)

However, some respondents rated evidence to be of average importance. As noted by one respondent “…*not all decisions need evidence*…” (D10). Unfortunately, the respondent did not provide any examples of such decisions.

Another parameter which also received mixed evaluations was “Improved fairness”. Most of the respondents thought it was very important to assess if a priority setting process was fair*;*

*”… Fairness is important in the pursuit of good governance, human rights, equity and democracy in decision-making especially in provision of public goals like health…”(D2)*

However, one respondent thought it was not very important if the outcomes are unfavorable; “…*a fairer process may not lead to better health outcomes*…” (D2)

There were also discrepancies in participants responses about the importance of reduced disagreements as a quality indicator. Some participants thought it was not an important parameter, explaining that disagreements could arise due to other reasons that may not be related to the priority setting process itself; while others thought that disagreements could be a healthy part of the process:

“…*Stakeholders may disagree and yet the priority setting process may be successful; disagreements are part of the process*…”(D12)

### National Responses

Almost all respondents (20/23) indicated that all the parameters in the framework are very important with the exception of “availability of incentives” where 16/23 indicated that it was very important. Specifically, increased efficiency, more appropriate allocation of resources, use of an explicit framework, increased use of evidence and fairness were all thought to be very important when evaluating priority setting. Many respondents identified the relationship between the parameters and the need for them to be concurrently evaluated. For example, fairness of the process was thought to be a very important parameter independently. However, participants also linked fairness to the use of evidence, equitable stakeholder participation and buy-in, which they thought to be important for implementation. Commenting on the link between fairness and the use of evidence, one respondent said:

*“So if the priority setting process follows the evidence I would say it is fair. If you didn’t follow any form of evidence then there is no way you can say whether it is fair or not fair …”(N13)*

Not only did respondents think evidence was relevant to fair priority setting, the majority of respondents also perceived the used of evidence as a very important parameter for evaluating priority setting: “*If you define your priority based on evidence then you are bound to succeed”* (N2).

Furthermore, all national level respondents rated all the stakeholder related parameters such as participation, understanding and compliance as “very important” both independently as well as through contributing to a fair priority setting processes. For example, one respondent linked fairness to equitable stakeholder participation since in a fair process the views of “weak” populations, which are often neglected, are considered. Furthermore, stakeholder participation was linked to stakeholder understanding and satisfaction. Respondents explained that an understanding of the process was important to ensure that stakeholders can participate fully and effectively, without limitations; which in turn would contribute to their satisfaction with the process. Some respondents thought that stakeholder satisfaction with the process encourages stakeholders to support the implementation of priorities and become true participants in working towards a common goal. According to one respondent, there has been a move to encourage greater participation of a broad representation of stakeholders in the priority setting process in Uganda, as seen in the last Health Sector Strategic and Investment Planning (HSSIP).

Parameters such as improved internal and public accountability, increased public confidence in the system, reduced resource wastage, reduced corruption, meeting institutional objectives, increased investment and strengthening of the health sector were all evaluated as very important.

Moreover, many respondents saw them as being interlinked. For example, increased investment in the health sector and strengthening of the health care system were considered to be very important in assessing how resources are mobilized and allocated as priorities are rolled out. Participants commented that if this is happening, the health system will remain strong, however, if investment in the health system decreases, the system will become fragmented. Hence, these thought that aligning resource allocation with ministry of health goals to be critical to successful priority setting.

*“So ensuring that finances go to health in sufficient levels to attain the goals is in fact the utmost priority. It is the final assessment of whether you have aligned all the resources correctly because basically you are giving value to all inputs and converting it into money”(N15)*

Similar to the district respondents, all respondents evaluated all the contextual factors (cultural, social, political and economic) as very relevant when evaluating priority setting, explaining that priority setting does not occur in a vacuum.

*“…Yes, contextual issues are benchmarks for policy setting. You cannot set your policy from nowhere. You need to know what are the economic issues, what are the political issues, what are the social issues, what are the…those parameters are very important for you to make policies and programs that address them, either in short term, medium term or long term..”(N8)*

### Parameters with mixed responses

While almost all respondents consistently rated all the parameters as very important when evaluating priority setting, 1–6 respondents thought some of the parameters were either of average importance and 1–2 evaluated them as not important. For example, one respondent cautioned that in a focus on efficiency as a parameter, it was possible to forget effectiveness;

*“…The overriding concern in social policy is more about fairness, social justice, and equity. Efficiency on the other side is not about effectiveness, it is about the highest return on each dollar invested…*” (N15)

Six respondents thought incentives were not very important because they could introduce bias or indicate a problem with the priority setting process whereby people have to be “convinced” to comply. In addition, some respondents did not evaluate reduced disagreements as an important parameter when evaluating priority setting because they argued that a reduction in disagreements does not necessarily mean that the result of the process is the best one, since disagreements can have a positive outcome. Another participant described how a lack of disagreement was not necessarily a good thing, since agreement could result from political coercion and does not mean that a decision was made based on the best evidence

*“…Disagreements especially functional ones are healthy and constructive”(N9)*

“*You may all be agreeing on something that is technically wrong … You may agree…the dominant figure, the President may want something that everyone will have to prioritize and implement even though they all don’t think it is right. But it will be implemented and have its effects just because of the political backing…*” (N1)

Furthermore, respondents highlighted the importance of parameters related to responsible use of the available resources;

“…*You may have a very beautiful plan, very technically sound, everyone is behind it but the systems that check corruption and everything are not there…”(N8)*

This might end up making priority setting fail.

The above responses from the respondents at the national level who rated some parameters as less important than others point at the need for a holistic evaluation, concurrently considering the different related parameters.

### Global responses

The majority of the global respondents evaluated almost all the parameters as very important with the exception of a couple that we discuss at the end of the section. Specifically, all parameters related to the priority setting process, for example “the use of evidence”, “a fair process” and “increased efficiency”*,* were evaluated as very important in evaluating the priority setting process. For example, commenting on the importance of improved efficiency, one respondent said it was the “*sine qua non of priority setting*”. Others explained that efficiency was important in a context of limited resources, noting that; “…*If the level of effort of conducting the priority setting is high* vs. *limited impact then it isn’t that efficient a process..”(G1).*

Furthermore, respondents thought that “more appropriate priority setting” was very important;

“*This is a key outcome and supports operational planning at a later stage”*. However a couple thought “appropriateness” may be difficult to assess given that priority setting is a political process.

Another parameter that was evaluated as very important was” use of evidence”. Respondents regarded it as fundamental in reducing arbitrarily and self interest. They indicated a preference for locally generated evidence, although they also gave caution with regards to the potential costs of collecting the evidence and that lack of evidence should not prohibit *“making hard decisions”.*

*“*Improved fairness” (of the priority setting process) was also evaluated as very important.

Respondents indicated that this parameter would be relevant in ensuring equity, although a few raised concerns with regards to lack of universal definitions of fairness.

All s*takeholder and public related parameters*, with the exception of reduced disagreements, were evaluated as very important in assessing the success of priority setting. For example, participants linked stakeholder understanding, satisfaction and compliance in their qualitative comments. In particular, respondents thought understanding was critical and would improve satisfaction, acceptance and confidence in the process; which would facilitate implementation.

Most of the respondents evaluated the parameters related to the priority setting institution as very important. For example, several explained that institutional capacity is fundamental to process rigor; while others explained their response with regards to the “proper allocation of resources”, linking institutional capacity to improved efficiency of the process. These participants perceived increasing institutional capacity as the primary goal of priority setting and they also linked it to increased investment in the health system;

*“…If we utilize a priority setting process efficiently, it should lead to efficiency gains and automatically create a 'push' mechanism with policy makers and ministries of finance to create improved & potentially increase resource allocation for health…”(G10)*

Similar to the global and national respondents, global respondents evaluated all contextual factors as very important to consider when evaluating priority setting.

### Parameters with mixed evaluations

Three to six respondents indicated that availability of incentives, reduction in disagreements, reduction in corruption, increased public knowledge and increased investment into the health sector were not important parameters when evaluating priority setting. While some respondents thought that availability of incentives was a very important parameter to ensure compliance since “*This will determine utilization and institutionalization of the process”*, others highlighted the way that using incentives may not be possible in all programs, and thus doing so may have detrimental impacts on other programs where incentives may not be available. Again some thought that the use of incentives was part of a separate step (implementation) and not part of the priority setting process; *“…Incentives for implementers may be useful in some instances, but do not determine whether priority setting is useful…”(G8)*

Furthermore, while most of the global respondents thought reducing disagreements was very important because it would be an indicator for assured continuity and support for the priority setting process, six respondents rated it as not important. Similar to respondents at the district level, these participants argued that disagreements are not necessarily bad or an indicator of poor processes, but may be a healthy part of priority setting, and could, in fact facilitate improvement and innovation in priority setting. For example one respondent stated that *“… disagreements encourage process improvement and innovation…” (G1)*

Responses were also divided with regards to some of the Institution and Health system related parameters. For example, some respondents evaluated reduction in corruption as not very important. These felt that it would be difficult to evaluate because, “…*What counts as corruption is complicated and what looks like corruption may be crucial to the success of the priority setting…*” (G11). Furthermore, a few respondents thought that it is not very important, (at the global level) to assess if the priority setting process aligns with institutional objectives and goals.

Particularly, some participants explained that ensuring alignment with institutional objectives and goals may not be the primary objective of priority setting; however, these respondents thought that it might be in the interest of the Ministry of Health if the priority setting process was aligned with the MOH goals.

### Cross-level comparisons

Respondents across all levels of decision-making validated most of the parameters proposed in Kapiriri and Martin’s framework as “very important” for evaluating priority setting. They also emphasized the interconnectedness between the success of one parameter and another. In many cases, disagreement on the importance of one parameter was due to the need for a holistic assessment of the situation at hand.

The parameter that received the lowest support across all the levels of decision-making was “availability of incentives to comply”, with less than 50% of the respondents at the global level, 67% of the national and 61% of the district level respondents rating it as very important, mainly because of the perception that incentives may be an indication of a weak priority setting process and they might lead to bias. This was the only parameter that was rated poorly among the national level respondents. The only other parameter that fewer respondents at all levels indicated was less important was “reduced disagreements”. Interestingly, at all levels, respondents gave the same reason for ranking this parameter as less important: that the disagreements may be a healthy part of the priority setting process.

While most of the “stakeholder” related parameters were deemed very important by almost all district and national level respondents, some global level respondents [4/17] consistently rated them as not very important. This pattern was followed in global respondents’ evaluation of ‘increased investment’, ‘strengthening of the health care system’, ‘contribution to institutional objectives’, ‘increased stakeholder understanding’, ‘use of explicit priority setting framework’ and public knowledge. Respondents at the global level did not provide qualitative explanations for their rankings. For example, while only 4/17 respondents at the global level thought that increased public knowledge was a very important indicator; compared to all respondents at the national and district levels. While respondents at the global level did not provide quantitative explanations, the few district level respondents who thought it was not “very important” explained that since the literacy levels of their population was low, improving the public’s understanding of priority setting may be difficult, and may not be a genuine reflection of the priority setting process.

### Validation of the feasibility of the means of verification

In this section of the paper, we present findings from the respondents’ assessments of how feasible it would be to collect the information needed to verify the above parameters. In addition, to test the validity of the responses, we pilot tested the ease of using the proposed means of verification to collect the relevant information at the national level. The main means of verification of the parameters were; (i) Observation at meetings, (ii) Interviews (exit and public surveys) (iii) review of documents including: popular media (newspapers), policy documents, and relevant publications.

### Findings from the survey

#### District assessment

*Meetings:* Most of the respondents indicated that it would be feasible, and easy to obtain permission by anyone who would be interested in observing at decision-making meetings. However, they indicated that information on the efficient use of time and the “use of evidence” might not be easy to assess at these meetings, which was surprising. The difficulty in assessing if evidence is used in the priority setting process, especially at the district level could partly be explained by participants’ responses that some of the necessary evidence used, for example, BOD information, could not be generated at a district level. Furthermore, participants indicated that data on whether or not time is used efficiently during a decision making meeting is not routinely collected at the district level; however, some respondents indicated that this could easily be computed by noting the start and end time of the meetings.

*Documents:* Most respondents indicated that the documents, including meeting minutes, budget reports, and hospital records would easily provide the relevant information, and could easily be accessed by anyone interested in reviewing them. Some, however, did not think it would be easy to access information on the number of appeals made, the degree to which the process is perceived to be fair, the number of complaints and the percent reduction in inequalities in health outcomes. This was because this information is neither routinely documented nor collected within the district.

*Interviews: Survey;* Several participants indicated that stakeholder surveys could not be easily administered within the district. This was, for the most part, attributed to the low literacy rates among stakeholders. Others attributed it to potential costs and human resources that might be needed to conduct the surveys.

*Exit interviews*: Respondents thought this was very feasible.

### National level assessment

*Meetings:* The responses with regards to the feasibility of a researcher attending meetings were mixed. Almost all the respondents thought it was feasible; however, they emphasized that there are bureaucratic procedures that have to be adhered to. First, it was emphasized that access depended on the level and stakes of the meeting. Senior management meetings as well as technical working group meetings, which involve the program leads within the ministry of health were thought to be relatively easy to access. However, participants explained that in order to attend the meeting, permission must be sought from the ministry of health permanent secretary as well as the secretary of the meeting. High-level meetings, for example, The Health Policy Advisory Committee which involves all health development partners that support the health sector, were thought to be less accessible. Among respondents, the perception was that while one could seek permission to attend high- level meetings, it might be difficult to obtain. Some participants also commented that high-level meetings are often inefficient, due to poor attendance and organization. One respondent intimated that high level meetings may not be that relevant to access since; *“most of the important decisions are not made within the meetings”* (N2). The respondent further explained that the technical decisions are made in the senior management meetings and the high level meetings often endorse these decisions.

*Documents:* Respondents thought most of the necessary information could easily be accessed through document review. Specifically, they mentioned the Health Sector Strategic and Investment Plan and budget documents, the Demographic Household Survey and special reports. Some health information is also reported in the newspapers. While they acknowledged that some of the specific information may not be routinely collected, such as numbers of stakeholder interactions, number of decisions made on time, and number of decisions appealed, they thought these could easily be added to the current information collection strategies. They also thought that if meetings were not accessible, it would be feasible to access the minutes when the meeting proceedings are recorded. However, several reported that they may not be aware of the commissioned studies because they may not be well documented, while others talked about the fact that data or evidence on which to base priority setting decisions is not always available.

*Interviews: Surveys;* Respondents thought that gathering information about stakeholder participation and satisfaction with the priority setting process was important. Currently, this is not done, but respondents believed that it should be done. However, the majority thought that conducting stakeholder surveys would be difficult due to logistical challenges and the associated costs. Hence, they thought that only political will would ensure that stakeholder surveys are conducted. Interviews with stakeholders, especially exit interviews after meetings, were suggested as the easiest means of conducting these surveys. However these interviews would be dependent upon the level at which the stakeholder was at: “*At the low level, yes. The community and stuff like that they always happen but these elites, you know, it’s not easy.”* (N7). There was also the concern that, even if interviewed, stakeholders may not be willing to share all of their views: *“…so what you may end up knowing is what they end of sharing with you. What they haven’t shared with you, you may not know” (N1).* This is not peculiar to Uganda but reflects a general limitation of interviews as an information gathering strategy.

*Exit interviews*: Respondents thought this was very feasible.

### Global level

*Meetings:* Most respondents thought it would be somewhat easy for a researcher to gain access to meetings, but as discussed at the national level, most thought it would depend on the level of the meeting and the type of issue under consideration. For example, meetings about sensitive issues such as limited resource allocation might not be accessible. Respondents suggested conducting exit interviews in addition to the meetings. *Documents*: Generally, the feasibility of accessing documents was seen as context-dependent. For example, some participants at the global level thought that access would depend on the level and the confidentiality attached to the meeting. Respondents commented that to access documents generated from a confidential meeting, approval would be required. Documents detailing sensitive information, for example evidence of corruption or financial issues, were also thought to be difficult to access. Most respondents felt that the easiest documents to access might be health records and reports on mortality and morbidity data, but were unsure how helpful these would be in assessing the rigor of the priority setting process. They raised concern with regard to the availability and accuracy of the facility based data in Low income countries; “*Quality and availability issues depend on the country and health system – for Low income countries this proves to be a continuous challenge*” *(G1).*

*Interviews: Surveys*; Most respondents believed that it would be somewhat easy to conduct surveys, but cautioned that surveys would require government and MOH facilitation. *Exit interviews*: Respondents, even before asking them about exit interviews recommended that meeting observations should be supplemented with exit interviews. They thought this was very feasible, these were ac.

### Piloting the means of verification

We piloted all the proposed means of verification in a real life context in practically evaluate the feasibility of the strategies proposed in the framework.

*Meetings:* The “outsider” research assistants were only able to attend the Joint Review Mission meeting which convened stakeholders to deliberate on the progress made on implementing the identified priorities and plan for future priorities. They were also able to conduct exit interviews. They however, failed to access any other meetings.

However, when we trained another research assistant who was already attending the meetings to become a participant observer, they were able to collect the necessary information from several decision making meetings.

*Documents:* The research assistant was able to secure all the relevant policy documents and meeting minutes. He was also able to access the newspapers in the review period. However, some of the statistical information was not well segregated, and the newspapers mostly presented information on new developments in the health sector, for example, funds allocated to districts, the introduction of new vaccines, and “scandals”; these included news stories on the embezzlement of funds and unacceptable maternal deaths. There were no examples of cases in which the rationale for the allocation of resources was provided to the public. Neither was there information on cases where the different programs or districts disagreed with the decisions made at the national level, and appealed the allocation decisions.

*Surveys;* From reviewing the minutes from the meetings, it was possible to assess when any kind of evidence was brought to the meeting and used to guide the discussions. For example, at one meeting, results from a stakeholder satisfaction survey were presented. Beyond those, the research assistant found it difficult to access information on the relevant studies that responded to the questions of interest, since these are managed in a separate division which is not integrated into the ministry of health and are seldom published. Due to resource limitations, it was not feasible to conduct a public survey to assess their knowledge of priority setting.

*Exit interviews*: This was very feasible. We were able to interview the targeted number of a wide selection of stakeholders who participated at the various decision making meetings.

### Cross- level comparison of the means of verification validation

There was some consistence in the evaluation of the means of verification proposed in the framework. Respondents at all levels indicated that exit interviews would be feasible and a credible approach to assessing the degree to which meeting participants were involved and were satisfied with the process. Other than the district respondents who all indicated that meetings would be easily accessible, both respondents at the national and global levels indicated that depending on the context, very high level meetings about sensitive issues may not easily be accessible. These findings were corroborated during the pilot testing.

While respondents at the global level thought it would be easy to conduct public knowledge and satisfaction surveys, respondents at the national and district levels indicated that lack of resources and low literacy levels may impact the feasibility of conducting surveys.

There was also consensus with regard to documents. While respondents indicated that it would be easy to access the relevant documents, including meeting minutes, there was concern that since most of the information that should be obtained from the documents may not routinely be collected. There was also concern that some of the available information tends to be incomplete.

## Discussion

To the best of our knowledge, this paper presents one of the first attempts to test the validity of a framework with the relevant stakeholders in an low income country context, at different decision making levels. Almost all the parameters identified in the framework were validated as very important when evaluating priority setting by over 90% of the respondents at the global, national and district levels. This is not surprising and could partly be explained by the methods through which the parameters were identified. These were based on the literature, experience as well as interviews with experts in priority setting in Low income countries [20].

While only a few respondents thought that two parameters – “use of incentives “and “reduced disagreements” – were not important, the logic behind participants’ explanations is interesting and hence worth discussing. Research supports the use of incentives to ensure that implementers comply with the agreed upon practices. For example, financial incentives have been used to improve health outcomes and quality of care [22, 23], to improve uptake of health programs in Low income countries [24]. However, a few of our respondents felt it would introduce bias in the decision-making process and that a good process would not require incentives. It is possible that respondents perceived incentives in terms of ‘financial’ pay offs. However, as one respondent pointed out, participation in the priority setting process should be viewed as an incentive for stakeholders to comply. In addition to legitimating the decisions, increased compliance with the decisions is one of the justifications for meaningful stakeholder engagement [21].

‘Reduced disagreements’ was another parameter that deserves further reflection. Respondents gave varying explanations as to why reduced disagreements may not be a very important parameter. We argue that in the context of democratic participation (which we believe should be the norm for good priority setting); while disagreements should be acceptable in the initial period, these should reduce, with time, as people are meaningfully engaged and agree and contribute to the priority setting decisions. This is why the framework proposes that this be evaluated as a “delayed parameter” i.e. after 2–3 priority setting cycles [20]. The thinking is that with an agreed upon explicit and fair process and criteria, even the ‘losers’ would be satisfied and not disagree with the decision [21].

The finding that there were no significant differences in the validation of the different parameters between the district and national levels may be an indication of the flexibility and or comprehensiveness of the framework. It is, however, important to consider why some respondents at the global level deemed some parameters as less important. While respondents’ lower rating of certain parameters, for example,” increased investment in and strengthening of the health sector”, and “increased public confidence in the health sector”, were surprising, these ratings could be attributed to the fact that many of these parameters could be affected by many external [contextual] factors beyond the priority setting process. The relatively low rating (8/17) of the other parameters such as ‘increased public knowledge’ and ‘stakeholder understanding’ by respondents at the global level could also in part, be explained by their level of decision-making.

These populations may be difficult to define at the global level.

The responses to our validation of the means of verification proved that these are easily feasible, with the exception of observation at meetings. The perception that an “insider” would be more likely to access meetings at the national level points to the common anxiety around external evaluation. Furthermore, it may affect the dynamics of the meeting. Arguably, if the framework is central to the planning cycle and routinely internally- as is advised; external evaluations may not be that intimidating. The consensus that not all the necessary evidence could be easily accessed may be explained by the jurisdictions of the different offices. However, since all health related studies are cleared by the same ethics body which requires researchers to articulate strategies for knowledge transfer; there is need for better tracking and synthesis of this up to date information so that it is more easily accessed by decision makers and researchers. An evidence-clearing house could facilitate this [25].

The other means of verification whose feasibility was questionable were the stakeholder surveys.

While we were unable to pilot test the feasibility of conducting surveys, respondents thought it would be feasible, resources permitting. They emphasized that this is one of the topics that the ministry of health was interested in, as was evidenced by the study those results were presented at one of the meetings during the course of the project. It was not clear if the ministry of health was interested in the priority setting related themes that the proposed survey would collect, since the survey they referred to related to public satisfaction with the services. However, given this interest; it is possible that these surveys could be integrated so as to evaluate not only the impact of the decisions but also stakeholder satisfaction with the priority setting process.

To the best of our knowledge, this is one of the few papers that report finding from a framework validation process, before its actual application. Similar to planning, lack of contribution to the development of tools and frameworks may sometimes lead to poor uptake and sustained utilization of the tools/framework. This approach provided the potential users of the framework with an opportunity to contribute to its refinement. Such a strategy fosters stakeholder understanding, capacity strengthening, and a sense of ownership of the framework. Furthermore, inviting users to contribute to the refinement of a framework they are expected to use is empowering, builds their confidence in their ability to apply it, and increases the potential for its use by these people [26].

### Limitations

Since the data presented here was basically collected quantitatively, not all respondents provided explanations for their responses; this limited our understanding of the reasoning behind their evaluations. Furthermore, purposeful sampling may have introduced bias and limit generalizability. However, since the focus was on validating a priority setting framework, it was reasonable that we involve people with the required knowledge and experience.

## Conclusions

This paper presented findings from a priority setting evaluation framework validation at the global, national and sub- national levels. We found that over 90% of the respondents at the three levels validated all but two of the parameters as very important when evaluating priority setting in Low income countries. Furthermore, all the means of verification for the parameters were largely validated as feasible, even within Low income countries.

Given the comprehensiveness and the number of parameters in the framework, it is important that policy makers integrate evaluation mechanisms into their priority setting processes from the start. This would not only ensure that evaluation actually occurs but would also facilitate ongoing reflection, and collecting of routinely relevant information that would have been otherwise ignored and forgotten by the time a summative evaluation is planned. Given the negative connotations attached to evaluations, we propose that the framework be used by the policy makers and other stakeholders who are involved in priority setting, as part of their planning process within the decision-making institutions so as to facilitate ongoing reflection and devising of improvement strategies. This will promote learning organizations and contribute to strengthening of institutional capacity for priority setting. Given the contradictions about some of the parameters by respondents at the global level, we would propose that if this framework is used at the global level, these few parameters should be discussed to allow the stakeholders involved in the process to decide whether or not they want to consider them.

Revisions to the framework included omitting the two parameters which were evaluated by the participants as not important to evaluating priority setting: availability of incentives to comply, and reduced disagreements from the framework. Furthermore the parameters were re-organized to reflect the order of their occurrence (referred to as domains): 1) The priority setting context, 2) The pre-requisites, 3) The priority setting process, 4) Implementation, 5) Outcome and impact (Table 4).

**Table 4:** Chronologically organized parameters of successful priority setting

Domains	Parameters of Successful Priority Setting
Contextual Factors	Conducive Political, Economic, Social and cultural context
Pre-requisites	
	Political will
	Resources
	Legitimate and Credible institutions
	Availability of incentives
The Priority setting process	
	Stakeholder participation
	Use of clear priority setting process/tool/methods
	Use of explicit relevant priority setting criteria
	Use of evidence
	Reflection of public values
	Publicity of priorities and criteria
	Functional mechanisms for appealing the decisions
	Functional mechanisms for enforcement
	Efficiency of the priority-setting process
	Decreased resource wastage/misallocation
	*Improved internal accountability/reduced corruption*
	*Increased stakeholder understanding, satisfaction and compliance with the Priority setting process*
	*Reduced dissensions*
Implementation	Allocation of resources according to priorities
	*Improved internal accountability/reduced corruption*
	*Strengthening of the PS institution*
	*Impact on Priority setting institutional goals and objectives*
Outcome and impact	
	*Impact on health policy and practice*
	*Contribute to the achievement of Health system goals* *-improved population health* *-reduction in health inequalities* *-fair financial contribution* *-responsive health care system*
	*Improved financial and political accountability*
	*Increased investment in the health sector and strengthening of the health care system*

Key: Non- italics = immediate parameters; Italics = delayed parameters

Future research should focus on applying the validated framework to cases of low income country priority setting at the different levels of decision-making, for further validation in real life decision making. Furthermore, there is need for systematic evaluation of the degree to which stakeholder involvement in tools development and/or refinement facilitates their uptake and utilization of the tool. It is important to also evaluate the facilitators and barriers to use of credible tools in guiding and evaluating priority setting in low income countries.

## Additional file

Additional file 1: Tools used in the validation study. Check list used in the collection of both the quantitative and qualitative data. (DOCX 160 kb)
