# Supplementary material for: International validation of quality indicators for evaluating priority setting in low income countries: process and key lessons
Source: BMC Health Serv Res. 2017 Jun 19;17:418. doi: 10.1186/s12913-017-2360-7 (PMC5477252; doi:10.1186/s12913-017-2360-7)
Supplement: Additional file 1: — Tools used in the validation study. Check list used in the collection of both the quantitative and qualitative data. (DOCX 160 kb) [file 12913_2017_2360_MOESM1_ESM.docx]

Checklist for validating the framework for evaluating Priority Setting within the health system

# Personal Description

# 1. Which of the below options best describes your current position?

|  | Policy-maker |
| --- | --- |
|  | Health care provider |
|  | Researcher |
|  | In a health system support role |
|  | Other, please specify... ______________________ |

# Section 1: Parameters for Evaluating Priority Setting

For the following questions, please assess the degree of importance of the different components of the framework - on a scale of 1-3 (low-high) indicate how important the following parameters are when evaluating priority setting. Where possible please also provide a brief explanation of your response.

# 2. When evaluating any priority setting (PS) process, how important is it to assess if the priority setting improves the efficiency of decision-making process (e.g. there are more benefits in following priority setting compared to the time and human resource investments in the PS process)?

|  | 1 - low importance |
| --- | --- |
|  | 2 - average importance |
|  | 3 - high importance |

# 2a. Explain your response here:

# 3. When evaluating any PS process, how important is it to assess if priority setting leads to more appropriate allocation of resources ?

|  | 1 - low importance |
| --- | --- |
|  | 2 - average importance |
|  | 3 - high importance |

# 3a. . Explain your response here:

# 4. When evaluating any PS process, how important is it to assess if an explicit framework is used in the PS process?

|  | 1 - low importance |
| --- | --- |
|  | 2 - average importance |
|  | 3 - high importance |

# 4a. . Explain your response here:

# 5. When evaluating any PS process, how important is it to assess if evidence is used in the PS process?

|  | 1 - low importance |
| --- | --- |
|  | 2 - average importance |
|  | 3 - high importance |

# 5a. . Explain your response here:

# 6. When evaluating any PS process, how important is it to assess if the PS process leads to the fair allocation of resources?

|  | 1 - low importance |
| --- | --- |
|  | 2 - average importance |
|  | 3 - high importance |

# 6a. . Explain your response here:

# 7. When evaluating any PS process, how important is it to assess if there was consideration of incentives to ensure that implementers comply with the PS process and the PS decisions?

|  | 1 - low importance |
| --- | --- |
|  | 2 - average importance |
|  | 3 - high importance |

# 7a. . Explain your response here:

# 8. When evaluating any PS process, how important is it to assess if stakeholders are satisfied with the process?

|  | 1 - low importance |
| --- | --- |
|  | 2 - average importance |
|  | 3 - high importance |

# 8a. . Explain your response here:

# 9. When evaluating any PS process, how important is it to assess if stakeholders understand the PS process?

|  | 1 - low importance |
| --- | --- |
|  | 2 - average importance |
|  | 3 - high importance |

# 9a. . Explain your response here:

# 10. When evaluating any PS process, how important is it to assess if stakeholders comply with the explicit PS processes?

|  | 1 - low importance |
| --- | --- |
|  | 2 - average importance |
|  | 3 - high importance |

# 10a. Explain your response here:

# 11. When evaluating any PS process, how important is it to assess if priority setting contributes to a reduction in stakeholder and public disagreements?

|  | 1 - low importance |
| --- | --- |
|  | 2 - average importance |
|  | 3 - high importance |

# 11a. Explain your response here:

# 12. When evaluating any PS process, how important is it to assess if priority setting contributes to a reduction in misuse and misallocation of resources?

|  | 1 - low importance |
| --- | --- |
|  | 2 - average importance |
|  | 3 - high importance |

# 12a. Explain your response here:

# 13. When evaluating any PS process, how important is it to assess if priority setting contributes to improving internal accountability and reducing corruption?

|  | 1 - low importance |
| --- | --- |
|  | 2 - average importance |
|  | 3 - high importance |

# 13a. Explain your response here:

# 14. When evaluating any PS process, how important is it to assess if the people setting the priorities have the necessary capacity to set priorities?

|  | 1 - low importance |
| --- | --- |
|  | 2 - average importance |
|  | 3 - high importance |

# 14a. Explain your response here:

# 15. When evaluating any PS process, how important is it to assess if its outcomes contribute to the Ministry of Health achieving its objectives?

|  | 1 - low importance |
| --- | --- |
|  | 2 - average importance |
|  | 3 - high importance |

# 15a. Explain your response here:

# 16. When evaluating any PS process, how important is it to assess if priority setting results in increased Public confidence in the Priority setting institution, the Ministry of Health and acceptance of PS decisions?

|  | 1 - low importance |
| --- | --- |
|  | 2 - average importance |
|  | 3 - high importance |

# 16a. Explain your response here:

# 17. When evaluating any PS process, how important is it to assess if the PS process involved the public and/or considered public values?

|  | 1 - low importance |
| --- | --- |
|  | 2 - average importance |
|  | 3 - high importance |

# 17a. Explain your response here:

# 18. When evaluating any PS process, how important is it to assess if priority setting increases public awareness and knowledge of the need to set priorities?

|  | 1 - low importance |
| --- | --- |
|  | 2 - average importance |
|  | 3 - high importance |

# 18a. Explain your response here:

# 19. When evaluating any PS process, how important is it to assess if priority setting contributes to achieving STATED health system goals?

|  | 1 - low importance |
| --- | --- |
|  | 2 - average importance |
|  | 3 - high importance |

# 19a. Explain your response here:

# 20. When evaluating any PS process, how important is it to assess if priority setting improves public accountability?

|  | 1 - low importance |
| --- | --- |
|  | 2 - average importance |
|  | 3 - high importance |

# 20a. Explain your response here:

# 21. When evaluating any PS process, how important is it to assess if priority setting leads to increased investment in and strengthening of the health system?

|  | 1 - low importance |
| --- | --- |
|  | 2 - average importance |
|  | 3 - high importance |

# 21a. Explain your response here:

# 22a. When evaluating any PS process, how important is it to assess the impact of the political contextual factors on the PS process and outcomes?

|  | 1 - low importance |
| --- | --- |
|  | 2 - average importance |
|  | 3 - high importance |

# 22b. When evaluating any PS process, how important is it to assess the impact of the economic contextual factors on the PS process and outcomes?

|  | 1 - low importance |
| --- | --- |
|  | 2 - average importance |
|  | 3 - high importance |

# 22c. When evaluating any PS process, how important is it to assess the impact of the social contextual factors on the PS process and outcomes?

|  | 1 - low importance |
| --- | --- |
|  | 2 - average importance |
|  | 3 - high importance |

# 22d. When evaluating any PS process, how important is it to assess the impact of the cultural contextual factors on the PS process and outcomes?

|  | 1 - low importance |
| --- | --- |
|  | 2 - average importance |
|  | 3 - high importance |

# 22e. Explain your responses to the above 3 questions here:

# 23. In addition to the parameters identified above, are there other parameters you consider to be important when evaluating healthcare priority setting?

# Section 2: Means of Verification

The parameters in Section I above can be verified using different strategies. In Section II below, please assess the feasibility of collecting the different information necessary to evaluate a priority setting process.

# Section 2a: Meetings

# 23. How easy would it be to access and observe at PS meetings?

|  | 1 - not easy |
| --- | --- |
|  | 2 - somewhat easy |
|  | 3 - very easy |

# 23a. Explain your response here:

# 24. How easy would it be to access and observe at PS meetings in order to collect information on the proportion of meeting time spent on PS (improved efficiency)?

|  | 1 - not easy |
| --- | --- |
|  | 2 - somewhat easy |
|  | 3 - very easy |

# 24a. Explain your response here:

# 25. How easy would it be to observe at PS/decision-making meetings in order obtain information on the number of decisions made on time?

|  | 1 - not easy |
| --- | --- |
|  | 2 - somewhat easy |
|  | 3 - very easy |

# 25a. Explain your response here:

# 26. How easy would it be to observe at PS/decision-making meetings in order obtain information on the number of times evidence is used in the decision making process?

|  | 1 - not easy |
| --- | --- |
|  | 2 - somewhat easy |
|  | 3 - very easy |

# 26a. Explain your response here:

# 27. How easy would it be to observe at PS/decision-making meetings in order obtain information on the number of different stakeholders (including the public) attending meetings and the number of times they express their opinions?

|  | 1 - not easy |
| --- | --- |
|  | 2 - somewhat easy |
|  | 3 - very easy |

# 27a. Explain your response here:

# Section 2b: Review of Documents

# 28. How easy would it be to access documents in order to collect information on the framework used to guide the PS process?

|  | 1 - not easy |
| --- | --- |
|  | 2 - somewhat easy |
|  | 3 - very easy |

# 28a. Explain your response here:

# 29. How easy would it be to access documents in order to collect information on the criteria used to identify the priorities?

|  | 1 - not easy |
| --- | --- |
|  | 2 - somewhat easy |
|  | 3 - very easy |

# 29a. Explain your response here:

# 30. How easy would it be to access documents in order to collect information on the number of decisions appealed and revised?

|  | 1 - not easy |
| --- | --- |
|  | 2 - somewhat easy |
|  | 3 - very easy |

# 30a. Explain your response here:

# 31. How easy would it to access documents in order to collect information on cases where the decision-making process is not fair?

|  | 1 - not easy |
| --- | --- |
|  | 2 - somewhat easy |
|  | 3 - very easy |

# 31a. Explain your response here:

# 32. How easy would it be to access documents in order to obtain information on existing incentive mechanisms to facilitate implementation of the identified priorities?

|  | 1 - not easy |
| --- | --- |
|  | 2 - somewhat easy |
|  | 3 - very easy |

# 32a. Explain your response here:

# 33. How easy would it be for one to access documents in order to obtain information on the number of complaints, about the decisions made, by stakeholders?

|  | 1 - not easy |
| --- | --- |
|  | 2 - somewhat easy |
|  | 3 - very easy |

# 33a. Explain your response here:

# 34. How easy would it be for one to access documents in order to obtain information on the degree to which resource allocation is aligned with the agreed-upon priorities?

|  | 1 - not easy |
| --- | --- |
|  | 2 - somewhat easy |
|  | 3 - very easy |

# 34a. Explain your response here:

# 35. How easy would it be to access documents in order to obtain information on the number of times the organization health budget is re-allocated from less prioritized to high priority areas?

|  | 1 - not easy |
| --- | --- |
|  | 2 - somewhat easy |
|  | 3 - very easy |

# 35a. Explain your response here:

# 36. How easy would it be to access documents in order to obtain information on the existing strategies to collect relevant data, including commissioned studies as evidence to guide PS?

|  | 1 - not easy |
| --- | --- |
|  | 2 - somewhat easy |
|  | 3 - very easy |

# 36a. Explain your response here:

# 37. How easy would it be to access documents in order to obtain information on the number of times the financial resource allocation decisions are publicized?

|  | 1 - not easy |
| --- | --- |
|  | 2 - somewhat easy |
|  | 3 - very easy |

# 37a. Explain your response here:

# 38. How easy would it be to access documents in order to obtain information on whether there’s additional investments in the heath sector?

|  | 1 - not easy |
| --- | --- |
|  | 2 - somewhat easy |
|  | 3 - very easy |

# 38a. Explain your response here:

# 39. How easy would it be to access documents in order to obtain information on the number of corruption instances reported?

|  | 1 - not easy |
| --- | --- |
|  | 2 - somewhat easy |
|  | 3 - very easy |

# 39a. Explain your response here:

# Section 2c. Health records and reports

# 40. How feasible is it to access health records and reports to obtain the relevant information?

|  | 1 - not easy |
| --- | --- |
|  | 2 - somewhat easy |
|  | 3 - very easy |

# 40a. Explain your response here:

# 41. How easy would it be to access health records and reports to assess the percentage reduction in mortality and morbidity (disease burden), as a result of the PS process?

|  | 1 - not easy |
| --- | --- |
|  | 2 - somewhat easy |
|  | 3 - very easy |

# 41a. Explain your response here:

# 42. How easy would it be to access health records and reports to assess the percentage reduction of the difference in health outcomes between the upper and lower social- economic quintiles?

|  | 1 - not easy |
| --- | --- |
|  | 2 - somewhat easy |
|  | 3 - very easy |

# 42a. Explain your response here:

# 43. How easy would it be to access health records and reports to assess the percentage of poor populations with catastrophic health expenditures?

|  | 1 - not easy |
| --- | --- |
|  | 2 - somewhat easy |
|  | 3 - very easy |

# 43a. Explain your response here:

# 44. How easy would it be to access institutional documents to assess the percentage of institutional objectives met, that are attributed to the PS process?

|  | 1 - not easy |
| --- | --- |
|  | 2 - somewhat easy |
|  | 3 - very easy |

# 44a. Explain your response here:

# 45. How easy would it be to access health records and reports to assess any changes in the retention of health workers?

|  | 1 - not easy |
| --- | --- |
|  | 2 - somewhat easy |
|  | 3 - very easy |

# 45a. Explain your response here:

# Section 2d. Stakeholder survey

# 46. How feasible would it be to conduct regular stakeholder SURVEYS (including the public) to evaluate their experiences with priority setting processes?

|  | 1 - not easy |
| --- | --- |
|  | 2 - somewhat easy |
|  | 3 - very easy |

# 46a. Explain your response here:

# 47. How easy would it be for one to conduct/ access stakeholder surveys to obtain information on the percentage of stakeholders (including the public) reporting satisfaction with the priority setting process and decisions?

|  | 1 - not easy |
| --- | --- |
|  | 2 - somewhat easy |
|  | 3 - very easy |

# 47a. Explain your response here:

# 48. How easy would it be for one to conduct/ access stakeholder surveys to obtain information on the percentage of stakeholders that can articulate the concepts used in PS?

|  | 1 - not easy |
| --- | --- |
|  | 2 - somewhat easy |
|  | 3 - very easy |

# 48a. Explain your response here:

# 49. How easy would it be for one to conduct/access stakeholder surveys to obtain information on the percentage of stakeholders (including the public) who understand the need for PS?

|  | 1 - not easy |
| --- | --- |
|  | 2 - somewhat easy |
|  | 3 - very easy |

# 49a. Explain your response here:

# 50. How easy would it be for one to conduct/access stakeholder surveys to obtain information on the percentage of stakeholders with increased capacity to set priorities?

|  | 1 - not easy |
| --- | --- |
|  | 2 - somewhat easy |
|  | 3 - very easy |

# 50a. Explain your response here:

# 51. How easy would it be for one to conduct/ access stakeholder surveys to obtain information on the percentage of stakeholders (including the public) reporting satisfaction with the health care system?

|  | 1 - not easy |
| --- | --- |
|  | 2 - somewhat easy |
|  | 3 - very easy |

# 51a. Explain your response here:

# Thank you for your time!!!
